# Seeking consent for research with indigenous communities: a systematic review

**DOI:** 10.1186/s12910-016-0139-8

**Published:** 2016-10-22

**Authors:** Emily F. M. Fitzpatrick, Alexandra L. C. Martiniuk, Heather D’Antoine, June Oscar, Maureen Carter, Elizabeth J. Elliott

**Affiliations:** 1 Discipline of Paediatrics and Child Health, Sydney Medical School, University of Sydney, Sydney, Australia; 2 The Sydney Children’s Hospital Network (Westmead), 4 Governor Phillip Place, West Pennant Hills, Sydney, NSW 2125 Australia; 3 Sydney Medical School, University of Sydney, Sydney, Australia; 4 The George Institute for Global Health, Sydney, Australia; 5 Dalla Lana School of Public Health, University of Toronto, Toronto, Canada; 6 Menzies School of Health Research, Darwin, Australia; 7 Marninwarntikura Women’s Resource Centre, Fitzroy Crossing, Australia; 8 Nulungu Research Institute, The University of Notre Dame, Broome, Australia; 9 Nindilingarri Cultural Health Services, Fitzroy Crossing, Australia

**Keywords:** Ethics, Informed consent, Research, Indigenous, Aboriginal, Oceanic ancestry group

## Abstract

**Background:**

When conducting research with Indigenous populations consent should be sought from both individual participants and the local community. We aimed to search and summarise the literature about methods for seeking consent for research with Indigenous populations.

**Methods:**

A systematic literature search was conducted for articles that describe or evaluate the process of seeking informed consent for research with Indigenous participants. Guidelines for ethical research and for seeking consent with Indigenous people are also included in our review.

**Results:**

Of 1447 articles found 1391 were excluded (duplicates, irrelevant, not in English); 56 were relevant and included. Articles were categorised into original research that evaluated the consent process (*n* = 5) or publications detailing the process of seeking consent (*n* = 13) and guidelines for ethical research (*n* = 38). Guidelines were categorised into international (*n* = 8); national (*n* = 20) and state/regional/local guidelines (*n* = 10). In five studies based in Australia, Canada and The United States of America the consent process with Indigenous people was objectively evaluated. In 13 other studies interpreters, voice recording, videos, pictures, flipcharts and “plain language” forms were used to assist in seeking consent but these processes were not evaluated. Some Indigenous organisations provide examples of community-designed resources for seeking consent and describe methods of community engagement, but none are evaluated. International, national and local ethical guidelines stress the importance of upholding Indigenous values but fail to specify methods for engaging communities or obtaining individual consent. In the ‘Grey literature’ concerns about the consent process are identified but no solutions are offered.

**Conclusion:**

Consultation with Indigenous communities is needed to determine how consent should be sought from the community and the individual, and how to evaluate this process.

**Electronic supplementary material:**

The online version of this article (doi:10.1186/s12910-016-0139-8) contains supplementary material, which is available to authorized users.

## Background

There are an estimated 370 million Indigenous peoples residing in approximately 90 countries [1]. They are among the most marginalised of peoples, which has a substantial impact on their quality of life and health [2]. Many research studies have been conducted in Indigenous communities, however non-Indigenous researchers have not always addressed community priorities, nor collaborated in research with Indigenous people [3, 4]. Rather, researchers have often been perceived as doing research *on*, not *with* Indigenous people, with little regard to local cultural protocols and languages and without seeking consent from communities [3–6]. When approaching individual participants for consent to participate in research, researchers often don’t communicate in ways that can easily be understood [3–6]. Recent international guidelines for researchers working with Indigenous communities stress the need for a more culturally sensitive and collaborative approach [7–14]. By working along-side Indigenous researchers and in continuous consultation with cultural advisors, some non-Indigenous researchers working with Indigenous communities are striving to be more ethically sound and culturally conscious, however, the inequality persists [4, 15]. It should be noted that due to the diversity of the world’s Indigenous peoples [1], the United Nations has not adopted an official definition of ‘Indigenous’ [2] and in specific regions there may be a preference for other terms for example ‘Native Hawaiian’ or ‘Aboriginal’. In this paper we use the term ‘Indigenous’ when referring to these populations in the general sense and when reporting specify in publications will use the name referred to there. In this review we include publications that describe specific guidelines for working with Indigenous populations, which may not capture research in low to middle income settings where the majority of the population is considered to be Indigenous. This review also excludes reports that are not published in English.

The history of consent itself is interesting. One of the earliest proclamations for the right to consent in human experimentation was made in 1891 by the Prussian Minister of the Interior with regard to Koch’s so-called ‘remedy’ of tuberculin injections for the treatment of tuberculosis in prisoners [16]. Guidelines specified that ‘the remedy must in no case be used against the patient’s will’ [16]. An early record of an official consent form dates back to 1900, when research was conducted on to control the transmission of yellow fever from Cuba [17]. This was developed in response to popular resistance, published in Cuban newspapers, to Spanish immigrants being used as “Guinea Pigs” [17]. In 1927 Claude Bernard proclaimed that potentially harmful experiments should not be conducted on men ‘even though the results might be highly advantageous to science, i.e. to the health of others’ [18]. The Nuremberg Code was formulated in 1949 to protect against experimentation such as that conducted without consent on prisoners of World War II [19]. The term ‘informed consent’ as used today, was coined in 1957 during the landmark case of Salgo versus Leland Stanford etc. Bd Trustees [20] in the United States after a doctor failed to inform a patient of the potential risk of paralysis following a novel surgical procedure. The World Medical Association worked on setting up protecting the rights of research participants and developed the first edition of the Declaration of Helsinki published in 1964 [13]. Unethical research projects were relatively common. For instance, in 1966, Beecher’s landmark publication on ethical irregularities cited at least 22 unethical medical research projects [21]. Other research conducted in the United States that enrolled impoverished African Americans with syphilis from 1932 until 1972 failed to treat participants with penicillin, long after it was known to be a highly effective treatment [22]. Even recently, the way in which the Human Genome Diversity Project has been conducted has been questioned in places such as Australia, with concerns that there was a lack of respect for the cultural values of Indigenous people [23].

Time is rarely invested to ensure that vulnerable populations understand the information provided to them about a research study [24]. Minogue (1996) claims that modern research can be so complicated that even a fully competent individual is unable to give informed consent (as cited by [25]). Beyond consent, the issue of ownership of research findings requires consideration. With advances in biotechnology there are implications with regard to intellectual property rights, where companies are trying to patent genetic material of research participants for their commercial value [26]. The World Intellectual Property Organisation’s (WIPO) Grand Council of the Crees [27], endorsed the United Nations Declaration for the Rights of Indigenous Peoples (UNDRIP) [9], highlighting the importance of protecting the genetic resources, traditional knowledge and traditional cultural expressions of Indigenous people. Many countries supported these values but it was not until 2007 that colonised countries such as the USA, Australia, New Zealand (NZ) and Canada formally endorsed this policy, indicating that Indigenous people of these countries have been particularly vulnerable to denial of their human rights, including when it comes to participating in research and intellectual property.

In countries where ethical recruitment of participants for research requires consent forms and participant information statements to be written in “plain English”, many potential participants without English as their first language require an interpreter [28]. The interpreter faces the challenge of both explaining research jargon across a language barrier, and translating the cultural and scientific significance of the research [28]. English is often the second, third or fourth language for Indigenous people, especially in remote communities [5]. This makes it difficult for Indigenous participants to fully comprehend the “foreseeable risks” to which they are consenting [28]. Yet, even with these known challenges, Indigenous participants’ understanding of and preference for the consent process has rarely been investigated [5, 29, 30].

Our aim was to conduct a systematic review of peer-review and grey literature to identify studies and guidelines that describe specific methods used for seeking consent for research with Indigenous communities and potential individual participants. We also aimed to include studies that described and evaluated the process of seeking consent and its impact on promoting understanding of research, considering individual participant preferences and cultural beliefs.

## Methods

**Fig. 1:**
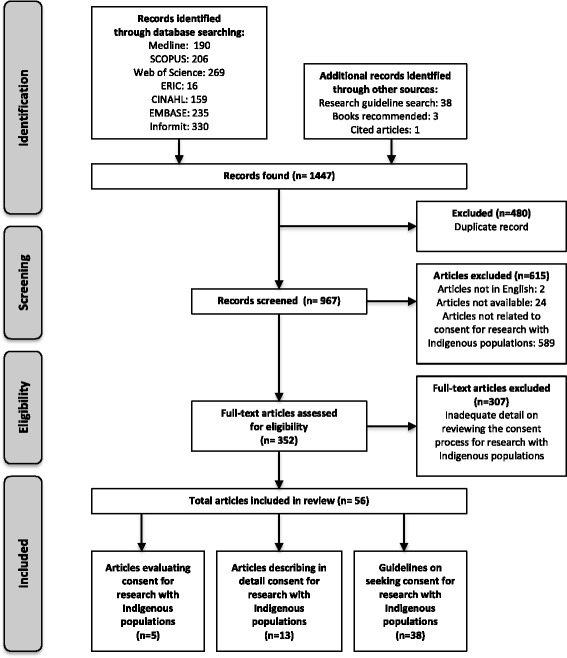
Selection Process for Publications Modified from the PRISMA 2009 Flow Diagram [32]

This systematic review was conducted and is reported in accordance with the PRISMA Guidelines [31, 32] (Fig. 1). With the support of Librarians of The University of Sydney Medical School, a search for publications was conducted using Medical Subject Headings (MeSH) such as ‘Informed Consent’, ‘Research’ and ‘Oceanic Ancestry Group’; and key words such as ‘Indigenous’ and ‘Aboriginal’ (and derivatives) which were combined in various ways depending on the functionality of the search engine (see Additional file 1). These were entered into medical literature databases: Medical Literature Analysis and Retrieval System Online (Medline); Scopus; Web of Science; Educational Resources Information Centre (ERIC); the Cumulative Index for Nursing and Allied Health Literature (CINAHL); Excerpta Medical Database (EMBASE); and Informit Indigenous Collection (IIC). In addition, we searched for resources including international ethical guidelines that describe the process of seeking consent for research with Indigenous populations. Due to the large diversity of Indigenous people around the world [1], and the lack of a single clear definition for an Indigenous population [2], we limited the search for research guidelines to international, national and local guidelines for colonised countries that held guidelines specifically for Indigenous populations and published in English. International guidelines that were included were sourced from websites of organisations such as the United Nations Declaration on the Rights of Indigenous Peoples (UNDRIP); World Health Organisation (WHO); World Medical Association (WMA); and the Council for International Organisations of Medical Science (CIOMS). National guidelines were identified through searching websites such as: The Lowitja Institute; the Australian Indigenous Aboriginal and Torres Strait Islander Studies (AIATSIS); and the Australian Aboriginal Health Info-net; the National Health and Medical Research Council (NHMRC) of Australia; the Health Research Council (HRC) NZ; the Canadian Institutes of Health Research (CIHR); and the National Institutes of Health (NIH), USA. In addition, we identified a number of guidelines that were state based, regional or locally created and which had a particular focus on seeking consent for research with Indigenous peoples. This search was conducted for the most recent and relevant publications from 1979 until November 2015. Articles that described in detail or evaluated the process of seeking informed consent for research specifically with Indigenous participants worldwide and published in English were eligible for inclusion. Articles were categorised into original research in which the process for seeking consent was evaluated; or which described in detail the process of seeking consent; or guidelines for ethical research (Fig. 1).

## Results

A total of 1447 articles was found. Of these, 1391 were excluded after abstract or full text review because they were duplicate publications; not published in English; not readily available; or did not evaluate or describe in sufficient detail the consent process for research with Indigenous people. There were 56 relevant research publications, 5 of which evaluated preference for method of delivery of information or understanding of information provided when seeking consent for research with Indigenous people (Table 1). We identified 13 publications that described methods for seeking consent (see Additional file 2). In addition, there were 38 guidelines for seeking consent for research with Indigenous people. These were categorised into international (see Additional file 3); national (see Additional file 4) and regional guidelines (see Additional file 5). We developed a checklist evaluating these guidelines (Table 2).

**Table 1:** Original Research Evaluating the Consent Process with Indigenous Populations

Citation (location)	Study Title	Study Description	Key Findings
Russell et al. 2005 (Alice Springs, NT, Australia) [33]	A pilot study of informed consent materials for Aboriginal participants in clinical trials	Information about an unlicensed vaccine trial for children was provided to 20 mothers who identified as Aboriginal and 20 non-Aboriginal mothers using verbal, written and visual materials. Preference and understanding was evaluated through a questionnaire.	▪ A single presentation to all participants was unlikely to enable informed consent; ▪ Participants identifying as Aboriginal preferred a flip chart with visual content presented by a doctor with an Aboriginal researcher present
Bull 2010 (Labrador, Canada) [34]	Research with Aboriginal Peoples: Authentic relationships as a precursor to ethical research	A researcher who identifies as Aboriginal and local to the community of Labrador interviewed 15 community leaders (11 leaders who identified as Inuit, Innu, or Inuit-Metis and 4 non-Aboriginal leaders nominated by the community) to investigate why authentic research relationships are important and how they are achieved with people who identify as Aboriginal.	To seek consent, the researcher must: ▪ Establish a reciprocal relationship with the community, involving them in the research process and interpretation of results ▪ Ensure the community understands the risks as well as the benefits of participating ▪ Respect cultural protocol ▪ Ensure the research is relevant to the community For this research project: ▪ Seek “collective consent” from communities ▪ Provide the option of oral or written consent for individual participants ▪ The individual consent process was not evaluated
McCabe et al. 2005 (Navajo Nation: Arizona, New Mexico & Utah, USA) [35]	The informed consent process in a cross-cultural setting: Is the process achieving the intended result?	The consent process for a diabetes education project with people of the Navajo Nation was analysed. Standardised consent forms in English were transcribed into a phonetic version of Navajo using the English alphabet. This was back translated to English and compared to the original English by a Navajo language expert. A second version was then tested with the Navajo Nation Medical Terminology Standardisation Committee. 333 Navajo people who do not use the written word were then asked to sign these forms by 4 Medical Interpreters who reflect on this process.	▪ Concerns were raised about this process by both participants and interpreters ▪ The consent process was contradictory; repetitive; untrustworthy; lengthy and discouraged participation Researchers conclude that consent forms should: ▪ Avoid legal and scientific jargon; ▪ Avoid repetition and wording that creates mistrust ▪ Have a logical flow of information when translated into complex languages Researchers should also: ▪ Ensure those seeking consent are culturally competent ▪ Avoid reliance on telephone communication ▪ Have community input into the consent forms ▪ Use graphics to illustrate concepts and assist understanding
Fong, M. 2004 (Hawai’i, USA) [36]	Native Hawaiian preferences for informed consent and disclosure of results from research	A survey was conducted with 429 Hawaiian participants (327 of whom were Native). The survey enquired whether participants would want to be asked for consent for the reuse of stored biological samples.	▪ Of native Hawaiian participants 77.9 % said they would want to be asked for consent for the reuse of biological samples if samples were identifiable compared to only 35–37 % if samples were anonymised Fong postulates that findings may be due to: ▪ Mistrust of research due to past discrimination ▪ Cultural beliefs, one which includes forbidding desecration of the placenta of the deceased
Baydala et al. 2013 (Alexis Nakota Sioux Nation, Alberta, Canada) [37]	“Making a Place of Respect”: Lessons learned in carrying out consent protocol with first nations elders	Research team members from the Alexis Nakota Sioux Nation and from the University of Alberta held 2 focus groups of 6 participants to reflect on a substance abuse prevention program for the youth of the Alexis Nakota Sioux Nation.	▪ Asking community Elders to sign a standard research consent form was considered insulting if they had already accepted a ceremonious offering of tobacco ▪ In response, researchers developed a new protocol to keep track of oral consent. This was approved by the ethics board, ensuring trust and respect of the community and integrity in research

**Table 2:** Evaluation of Current Guidelines for Seeking Consent for Research with Indigenous Populations

	CBD 2002 [7]	UN 2005 [8]	UNDRIP 2007 [9]	UN 2009 [10]	WHO 2009 [11]	UN 2013 [12]	WMA DOH 2013 [13]	WIPO 2015 [14]	NHMRC 2003 [49]	NHMRC 2005 [50]	CRC TS 2006 [51]	NHMRC 2007 [52]	CRIAH2008 [53]	CSIRO 2009 [54]	NHMRC 2010 [55]	NHMRC 2010 [56]	Lowitja 2011 [5]	AIATSIS 2012 [57]	NHMRC 2015 [58]	NZ Ministry of Health 2006 [59]	PWG 2010 [60]	HRC NZ 2010 [61]	NEAC 2012 [62]	NEAC 2012 [63]	AAND 2010 [64]	CIHR 2014 [65]	The Belmont Report USA 1979 [66]	TEC 2014 [67]	DK&CRC 2005 [68]	DK&CRC 2009 [69]	KLC 2011 [70]	KLC IP & TKP 2011 [71]	AH&MRC 2013 [72]	Ninti One 2015 [73]	UoVic 2003 [74]	Nuu-Chah-Nulth 2008 [75]	SNC 2014 [76]	Portland Area IHS IRB 2002 [77]
Guideline characteristics																																						
Indigenous research guildeline (versus General)		✓	✓		✓	✓			✓	✓	✓		✓	✓	✓		✓	✓			✓	✓			✓			✓	✓	✓	✓	✓	✓	✓	✓	✓	✓	✓
Notes that Indigenous people are a distinct group	✓	✓	✓	✓	✓	✓		✓	✓	✓	✓	✓	✓	✓	✓	✓	✓	✓	✓	✓		✓	✓	✓	✓	✓		✓	✓	✓	✓	✓	✓	✓	✓	✓	✓	✓
Embodies respect for Indigenous culture	✓	✓	✓	✓	✓	✓		✓	✓	✓	✓	✓	✓	✓	✓	✓	✓	✓	✓	✓	✓	✓	✓	✓	✓	✓		✓	✓	✓	✓	✓	✓	✓	✓	✓	✓	✓
Recommends Indigenous consultation in protocol development	✓	✓		✓	✓	✓		✓	✓	✓	✓		✓	✓	✓	✓	✓	✓	✓	✓	✓	✓	✓	✓	✓	✓		✓	✓	✓	✓	✓	✓	✓	✓	✓	✓	✓
Indigenous members on research ethics\advisory board					✓				✓	✓	✓			✓	✓		✓			✓	✓	✓	✓	✓		✓		✓	✓	✓	✓	✓	✓	✓	✓	✓		✓
Research team includes local Indigenous community members	✓			✓	✓	✓				✓	✓		✓	✓	✓		✓	✓	✓	✓	✓	✓	✓	✓		✓		✓	✓	✓	✓	✓	✓	✓	✓	✓	✓	✓
Reciprocity & benefit sharing with Indigenous community	✓	✓			✓		✓	✓	✓	✓	✓	✓	✓	✓	✓		✓	✓	✓	✓	✓	✓	✓	✓		✓		✓	✓	✓	✓	✓	✓	✓	✓	✓	✓	✓
Identification of ownership of data	✓	✓			✓	✓		✓	✓	✓	✓	✓	✓				✓	✓		✓	✓	✓				✓			✓	✓	✓	✓	✓		✓	✓	✓	
Research is identified as valued by the Indigenous community	✓			✓	✓		✓		✓	✓	✓		✓		✓	✓	✓	✓	✓		✓	✓	✓	✓	✓	✓		✓	✓		✓	✓	✓	✓	✓		✓	✓
Risks to the Indigenous community as a collective are explained	✓	✓		✓	✓			✓	✓	✓	✓			✓		✓	✓	✓		✓	✓	✓	✓	✓		✓				✓	✓	✓	✓					✓
An ethics committee has approved the project	✓			✓	✓		✓		✓	✓	✓	✓	✓			✓	✓	✓	✓	✓	✓	✓	✓	✓		✓	✓	✓	✓		✓	✓	✓	✓	✓	✓	✓	✓
Declare funding source to Indigenous communities	✓				✓		✓			✓	✓					✓	✓	✓	✓			✓	✓			✓			✓			✓					✓	✓
Recommendations regarding the consent process																																						
Indigenous community consent required	✓	✓		✓	✓	✓			✓	✓	✓		✓	✓		✓	✓	✓	✓	✓	✓	✓	✓	✓		✓		✓	✓	✓	✓	✓	✓	✓	✓	✓		✓
Indigenous individual consent is required	✓	✓		✓	✓	✓	✓		✓	✓	✓	✓	✓	✓		✓	✓	✓	✓	✓	✓	✓	✓	✓		✓	✓			✓	✓	✓	✓	✓	✓	✓	✓	✓
Free, prior and informed consent is sought	✓	✓	✓	✓	✓	✓	✓	✓	✓	✓				✓			✓	✓	✓				✓	✓	✓	✓			✓	✓	✓	✓	✓	✓			✓	
Waiver of consent may occur with ethics committee approval				✓			✓									✓			✓	✓			✓	✓		✓	✓										✓	✓
Signed consent is sought for individuals	✓			✓	✓		✓		✓	✓	✓	✓				✓	✓	✓	✓	✓	✓	✓	✓	✓		✓	✓		✓			✓	✓				✓	✓
Oral consent is possible for individuals					✓		✓		✓							✓			✓	✓	✓	✓	✓	✓		✓	✓										✓	✓
Full disclosure of risks and benefits of research provided	✓	✓		✓	✓		✓	✓	✓	✓	✓			✓		✓	✓	✓	✓	✓	✓	✓	✓	✓		✓	✓			✓		✓	✓	✓			✓	✓
Plain language explaination of research provided in writing	✓			✓	✓				✓	✓	✓					✓	✓	✓	✓	✓	✓	✓	✓	✓		✓	✓		✓	✓	✓	✓	✓	✓				✓
Access to an interpreter is provided				✓						✓							✓		✓	✓		✓				✓			✓	✓	✓	✓	✓					✓
Information provided in particpants language of preference		✓								✓							✓		✓			✓				✓			✓	✓		✓						✓
Suggests the use of appropriate visual aides for seeking consent											✓						✓									✓			✓									
Local Indigenous advice on consent materials provided																	✓					✓							✓			✓	✓					✓
Research information is provided on a number occasions				✓	✓					✓				✓	✓		✓	✓		✓	✓	✓	✓	✓		✓				✓		✓						
Adequate time is provided to review research information	✓	✓		✓	✓				✓	✓	✓			✓			✓	✓	✓		✓	✓	✓	✓		✓			✓	✓	✓		✓	✓		✓		
Opportunity is provided to address participant questions	✓			✓	✓				✓	✓							✓		✓	✓	✓	✓	✓	✓		✓	✓			✓								✓
Ensure consent is truly informed				✓	✓		✓		✓								✓					✓	✓	✓			✓		✓					✓				✓
Option to opt out or decline consent must be provided	✓	✓		✓	✓		✓		✓	✓	✓		✓	✓		✓	✓	✓	✓	✓	✓	✓	✓	✓	✓	✓	✓		✓	✓	✓	✓	✓	✓	✓	✓	✓	✓
Consent required for protocol changes	✓									✓	✓					✓	✓		✓			✓	✓			✓			✓	✓		✓	✓					✓
Confidentiality is discussed while seeking consent	✓	✓		✓	✓		✓	✓	✓	✓	✓	✓		✓		✓	✓	✓	✓	✓	✓	✓	✓	✓		✓	✓	✓	✓	✓	✓	✓	✓	✓	✓		✓	✓
Consent is sought to identify individuals if relevant												✓				✓	✓	✓	✓	✓	✓	✓	✓			✓	✓			✓		✓	✓		✓		✓	✓
Continued consent required throughout project	✓			✓	✓					✓	✓				✓	✓	✓	✓	✓		✓	✓		✓		✓			✓	✓	✓	✓		✓	✓			✓
Consent for reuse of data	✓				✓		✓			✓	✓					✓	✓		✓		✓	✓	✓	✓		✓	✓		✓			✓	✓				✓	
Tiered consent be obtained for future research	✓				✓					✓						✓			✓		✓	✓																
Broad consent be obtained for future research							✓		✓							✓			✓																			
Consent for dissemination of research	✓				✓			✓	✓	✓	✓	✓	✓	✓		✓	✓	✓	✓	✓	✓	✓	✓	✓		✓			✓	✓	✓	✓	✓	✓	✓		✓	✓
Feedback of research results to the communities	✓	✓			✓		✓		✓	✓	✓	✓	✓	✓	✓	✓	✓	✓	✓		✓	✓	✓	✓		✓		✔			✓	✓	✓	✓	✓	✓	✓	✓

### Original research evaluating the consent process

A total of five published research projects was identified which evaluated the preference for method of delivery of information or understanding of information when seeking consent for participation in research with Indigenous populations in Australia, USA, and Canada [33–37]. They emphasised the need for involvement of local Indigenous people in seeking consent, establishing good relationships between researchers and potential participants, cultural competence, and clear communication using plain language with visual cues (Table 1).

Of these five, only one study evaluated both preferences for method of delivery of information *and* understanding of information presented when seeking consent for research with an Indigenous population [33] (Table 1). This was a pilot study, set in Alice Springs in the Northern Territory, Australia, which was designed to evaluate mothers’ understanding of an unlicensed pneumococcal vaccine trial proposed for their children [33]. Twenty non-Aboriginal mothers and 20 mothers who identified as Aboriginal were recruited to the study. Eleven of the Aboriginal women did not speak English as their first language. Non-Aboriginal mothers had the project information explained to them by a nurse and were provided with ‘standard consent materials’ in the form of an information booklet containing only English text with time was allowed for questions. This process was evaluated by the non-Aboriginal mothers through a questionnaire. Aboriginal mothers had information presented to them by research team members – two of either a doctor, a nurse or a health worker who also identified as Aboriginal. The presentation was in plain English using a flip chart containing text and computer graphics. A 12-page booklet containing black and white diagrams and text was also provided [33]. Aboriginal mothers were then interviewed about the consent process with an Aboriginal health worker, supervised by the principle investigator. Responses were analysed by two researchers (their ethnicity was not specified) who rated their impression of the participants’ level of understanding of the consent information. Preference for consent materials and mode of delivery was also evaluated. It was concluded that a one-off presentation was inadequate to ensure informed consent from all participants.

Aboriginal participants preferred to receive research information from a doctor working together with an Aboriginal health worker using a flipchart rather than the information booklet [33].

### Articles describing the consent process in detail

We identified 13 articles from Australia, NZ, Paraguay, Canada, Malawi, Peru and the USA. These research publications describe a variety of methods used to aid communication during the consent process (Additional file 2) [4, 29, 30, 38–47]. Methods described include: using pictorial aids in a flip chart, presented with a local Aboriginal researcher to seek consent for research [30, 38, 45]. Some studies specify that an interpreter presents the research information in local Indigenous languages [29, 30, 38–41, 47] and some of these methods resulted in a high participation rates with 81–95 % [38, 42, 48], while others despite these efforts participation rates varied from 42–65 % [29, 44]. Authors who identify as Indigenous describe a more naturalistic approach to research, through an Indigenous research paradigm, embracing cultural protocol through practices such as storytelling [4, 41, 42]. Researchers only seek oral consent, for fear of creating a power imbalance between themselves and the Indigenous community when seeking written consent [43]. Community collaboration and consent is essential in order to prevent researchers who are not local to a community from making assumptions about how research and the process for seeking consent should be conducted [4, 30, 38–42, 45–48].

### Guidelines for research with Indigenous communities

International and national research guidelines have been produced by a number of government, health and research organisations. A total of 38 current guidelines that describe in detail methods for seeking consent for research with Indigenous populations is included (Additional files 3, 4 and 5). These are categorised as international (*n* = 8), (Additional file 3) [7–14]; national (*n* = 20) (Additional file 4) [5, 49–67]; and state, regional or local guidelines (*n* = 10) (Additional file 5) [68–77]. International guidelines were sourced from the United Nations (UN); World Health Organisation (WHO); World Medical Association (WMA) and the Council for International Organisations of Medical Science (CIOMS). National guidelines were sourced from Australia; NZ; Canada; and the USA. These guidelines espouse broad principles of research with Indigenous people including reciprocity, respect, equality, responsibility, survival and protection and spirit and integrity [49]. Many mention the need for both community and individual consent for research. Most advise researchers to seek free prior and informed consent from any potential research participant. Consent is to be voluntary, sought ‘free’ of coercion; with prior notification, with sufficient time for consideration and consultation. Consent is to be ‘informed’ with participants being made aware of both the benefits and risks of the project and their right to decline consent or to withdraw at any time. Details of the research must be explained to potential participants in plain language. Guidelines highlight Indigenous populations as vulnerable research participants and the potential for power imbalance. Few guidelines highlight the need for researchers to accommodate an oral tradition or cultural values. Most guidelines fail to recommend that researchers interpret study information into the participant’s first language. Few provide practical advice for seeking consent.

Specific advice is provided in individual guidelines. For example, in order to uphold the right to self-determination for Indigenous people [15] the NHMRC Values and Ethics Guidelines recommend that an Indigenous research project should be developed and initiated *with* community members, *for* community members and with the help of non-Indigenous research bodies only if required. This assumes that when working in a collaborative way, differences in culture and values may be resolved as Indigenous people help conduct the research. However, some authors caution that some individuals consulted by researchers planning a study may not necessarily be appropriate to give community consent if they are not well connected or respected by the local community and truly able to advocate for local needs [78]. Cultural awareness is key when seeking consent in Indigenous communities, and certain members of the community may be required to oversee proceedings. For example, when considering a project on vulvar cancer in women who identified as Indigenous Australians, the one female member of the local research council was absent [47]. As the subject of research was considered to be “secret women’s business” in the community’s culture, the committee felt it was not their place to approve the project [47].

When seeking approval for a project from the Western Australian Aboriginal Health Information Ethics Committee, researchers are required to explain exactly how they aim to uphold the six main values published in the NHRMC Values and Ethics Guideline [5, 49, 50, 58, 78]. A Values and Ethics Statement produced by the NHMRC describes in detail the values determined to be important to Australian Indigenous culture, namely: reciprocity; respect; equality; responsibility; survival and protection and above all states that the project be conducted in the right spirit and with integrity [5, 49, 50, 58, 79]. This protocol is helpful, however it does not specify how the process of seeking consent for research could reflect these values and leaves the interpretation up to the researcher. The NHMRC has recently revised their 2007 statement on ethical conduct in human research [58]. This specifies that researchers should engage and seek the support of a community when initiating a research project; acknowledge the distinctive language and culture of individual communities; involve Indigenous people in upholding these values in an equal partnership and with respect; and consider any local language and cultural protocols determined by the Indigenous communities involved in the research [58]. The new guideline recommends that researchers refer to the National Health and Medical Research Council Values and Ethics Guidelines, Australia [49].

The latest edition of the Australian Institute of Aboriginal and Torres Strait Islander Studies guidelines [57] include a section (Principle 6) that highlights the importance of consultation, negotiation and seeking free, prior, informed consent for research whether with Indigenous or non-Indigenous peoples. It outlines the preparations one should make prior to approaching an Indigenous community to seek their involvement in research and advise researchers to seek protocols from the local community. The AIATSIS guidelines [57] emphasise that it is important to seek permission from a community as a group as well as from individuals and to provide a plain English statement. They specify that researchers should identify the appropriate individuals with whom to consult initially, and should involve the Traditional Owners who speak for the Country. They emphasise that free prior, informed, consent should then be sought from all participants. The AIATSIS guidelines do not address the issue that not all Indigenous peoples use the written word, nor do they discuss circumstances in which English is an individual’s second or third language [57].

The Lowitja Institute (Australia) has published a book providing practical guidelines for research in Indigenous health [5]. The Indigenous authors describe culturally safe approaches to conducting research with Indigenous peoples. They emphasise that when seeking consent for research community leaders should first be approached for permission, and that the community should be consulted as a whole before researchers approach individual participants. Continual engagement with the community and its leaders and collaboration with Indigenous researchers throughout the project is highly recommended. This empowers people who identify as Indigenous, enabling the study to be culturally informed and increasing acceptance amongst the community. The guidelines are culturally sensitive, clear and applicable to research in Australia [5].

State, regional and local guidelines (*n* = 10) included in this review discuss similar values to those included in national and international guidelines [68–77]. These guidelines were difficult to source and it is likely that some are missed, including online guidelines and guidelines embedded in ethics protocols of universities and other community organisations.

### Evaluation of current guidelines for seeking consent for research with indigenous populations

In our search we found 4 review articles of guidelines for ethical research with Indigenous populations [80–83]. In some reviews, the attributes of each guideline are critically analysed and tabulated against a checklist of desirable qualities. These reviews are valuable in identifying guidelines although many are out-dated and not directly relevant to our review. Based on these reviews, we developed a checklist to assist in evaluating the quality of the 38 guidelines included in our review (Table 2).

Many of these guidelines assessed included general statements such as ‘the research ethics committee shall endeavour to protect the integrity of our Indigenous Knowledge, our culture and the members of the Six Nations from harm or abuse’ Six Nations Council [76]. However, guidelines do not specify how to achieve this. It did not meet criteria to be included under the checklist requirement in Table 2 for researchers to ensure ‘Risks to the Indigenous community as a collective are explained’. Guidelines do not always specifically translate research values into practice.

In regards to consent for re-use of data, some guidelines were vague, suggesting that broad consent be obtained for ‘future research’ [13, 49, 56, 58]. This may leave the future use of data open to the interpretation by the researchers, which historically, may not always have been done in an ethical way. In one such study with the Havasupi tribes in the USA, blood samples were collected for diabetes research in 1990 and these samples have been re-used over subsequent years without the community’s knowledge [84–89]. Scientists were using the tribe’s genetic material to examine links to schizophrenia as well as conducting other studies which the tribes felt questioned their sovereignty [84–89]. The remaining samples were later repatriated in a ceremony [88, 89]. Helgesson [90] argues that it was not the fact that broad consent was sought from the Indigenous community that caused the upset, but that the Havasupai tribe did not realise the implications of broad consent. That is, the research process, was not adequately communicated in the first place. It is essential to establish ethics committees with Indigenous members to monitor and approve future medical research projects because there is little protection in national and international law about genetic sampling protecting Australian Indigenous people [91, 92].

Most of the guidelines provide a general background of the principles important to uphold when conducting research with Indigenous populations, but practical specifications for engaging an Indigenous community or communicating with individual participants in order to seek free, prior, informed consent, are better garnered from individual research studies as described in Tables 1 and 2 [4, 29, 30, 33–48].

## Discussion

We found few publications that describe specific communication methods for seeking informed consent for Indigenous research and even fewer that evaluate participants’ understanding or preferences for the process [4, 5, 7–14, 29, 30, 33–77]. This may be explained by publication bias or simply reflect lack of attention to the consent process in these vulnerable populations [82, 93]. As mentioned previously there are over 90 countries with Indigenous populations [1], however this review is limited to reports published in English and might also exclude research from countries in which the majority of the population may considered to be Indigenous, but have not necessarily specified that they are working with an ‘Indigenous’ population.

The only original research study evaluating the consent process, Russell et al [33] had a very small study sample, with only twenty mothers who identified as Aboriginal. Researchers identified the need for multiple presentations of information, including by local Aboriginal people, and use of visual aids to enhance participants’ understanding of the proposed research. The role participants played within the Aboriginal community and the specific communities where the participants lived were not described, but it is unlikely that this is a representative sample of Aboriginal people living in Central Australia and hence the results may not be generalizable. Bull [34] did not evaluate understanding or preferences regarding the consent process by the community or individuals, but through discussions with community leaders concluded that researchers must establish a reciprocal relationship with the community, ensure that research is relevant to that community, respect cultural protocols and involve Aboriginal people in the conduct and reporting of the research. McCabe [35] describes a research approach that was badly received by the community and provides lessons about what to avoid, including reliance on telephone communication. The need for community input into consent forms, avoidance of scientific jargon, and inclusion of graphics were recommended to increase understanding [35]. Fong [36] assumes that Hawaiians’ failure to consent to use of their biological samples for future studies related to cultural beliefs relating to desecration of body parts but did not substantiate this theory. This illustrates the need for researchers to thoroughly understand cultural beliefs and practices. Baydala [37] used a novel consent process respecting local cultural protocol when seeking consent with elders of Aboriginal communities of the Alexis Nakota Sioux Nation, Alberta, Canada, but acknowledged that this process may be inappropriate among other First Nations or Inuit communities of Canada.

In articles that describe the consent process in detail, those with high recruitment typically involved local Aboriginal organisations and leaders in the project design and recruitment [38, 42, 48]. In The Lililwan Project, researchers employed local Aboriginal people as community navigators to disseminate study information, assist with recruitment of participants and support the research team in seeking written consent from parents and carers [38]. A plain English statement and consent form was read out and interpreted in their preferred language with the assistance of pictorial aides [38]. There was 95 % participation in this study, while using this method to seek consent [48]. In NZ there is a term Kaupapa Māori Research, which encompasses the idea that research with Māori populations should be driven by Māori researchers [4, 39]. Wilson [42] emphasises the importance of researchers fostering equitable relationships with the community they are working with, however some studies we found do not appear to involve local Indigenous community members in this way [29, 30, 43]. There is often a communication gap between Indigenous and Non Indigenous communities, especially between them and researchers and health services [94]. Alison Hoy [95] raised concerns that this may result in Indigenous people being excluded from crucial projects because of the perception of exploitation. This may deny Indigenous people the opportunity of helping address major health disparities and benefiting from the results. Teams of non-Indigenous researchers often fail to acknowledge their different cultural backgrounds and philosophies on life compared to Indigenous people [96]. Indigenous and Non-Indigenous authors compare and discuss how the differences between their “two worlds” impacts on the quality of research, noting that certain attitudes to research with Indigenous communities can prevent it from being conducted in a culturally respectful way [4, 5, 34, 35, 42]. There is a language of inequality embedded even within today’s publications, with the idea that Non-Indigenous people are researchers and Indigenous people are the researched [4]. It is thus necessary for researchers to engage a community and foster relationships with leaders and local organisations [97].

A total of 38 research guidelines were included in this review, the majority of which define the term free, prior and informed consent, but few of which describe how this can be achieved. Some guidelines do not mention Indigenous communities as a special group for consideration and simply state that “language and cultural” differences must be accounted for [13, 66]. Others include special sections emphasising the vulnerability of Indigenous people with regard to research [10, 12, 14] or remind us of Indigenous Peoples rights [8, 9, 11].

Some research guidelines note the importance of seeking consent for research initially from a community leader or council of elders [5, 7, 8, 10–12, 49–51, 53, 54, 56–63, 65, 67–75, 77]. Some determine that only the community has control over what information may be publicly released [8]. The concept of community consent is debated and many questions arise regarding what comprises a community and who can represent a community [72, 98]. Tsosie [99] notes that in some communities a member may hold the duty to keep secret certain knowledge even from other community members and that this may prevent provision of consent or dissemination of results.

Of the national guidelines (*n* = 20), NZ appears to offer the most protection for the rights of Māori, based on the spirit of the Treaty of Waitangi and Kaupapa Māori Research [59–63]. Culturally significant language is embedded into the research guideline, which identifies that it is preferable for Māori researchers to take charge of projects that are about or may affect Māori people [59–63]. Australia and Canada also have culturally sensitive guidelines but it is difficult to translate values espoused into practice [49–58, 64, 65]. Neither of the USA guidelines specifically addressed the consent process for research with Indigenous people [66, 67]. The Tribal Epidemiology Centres report that research organisations have found it difficult to work with people who identify as American Indian or Alaskan Native due to their mobility, literacy, and language barriers [67]. Community Based Participatory Research with locally designed protocols has increased participation rates [67].

## Conclusion

Researchers working with Indigenous people must be culturally respectful [92]; cognisant of the law [20, 26, 100]; and conscious of the impact of research on communities as a whole [101]. Ethical guidelines emphasise the need to approach Indigenous communities, governing bodies and leaders for consent prior to approaching individual participants. Best practice recommends a collaborative approach, in partnership with local Indigenous people, and research that addresses community priorities. Communication aids and involvement of local Indigenous researchers can help overcome language and cultural barriers when seeking consent [4, 5, 33–35, 38, 39, 42, 48, 65, 67].

Our literature review identified few original articles that evaluate preference or understanding of the communication methods used in seeking informed consent for research with Indigenous populations. To ensure that future research is of value to Indigenous communities, there is an urgent need for research to understand how to best seek consent.

## Additional files

Additional file 1: Table S3. Medical Subject Headings and key words used to search Medline. (XLSX 43 kb)
Additional file 2: Table S4. Research describing the consent process with Indigenous populations in detail. (XLSM 50 kb)
Additional file 3: Table S5. International guidelines for seeking consent for research with Indigenous populations. (XLSM 39 kb)
Additional file 4: Table S6. National guidelines on seeking consent for research with Indigenous populations. (XLSX 40 kb)
Additional file 5: Table S7. State/Regional/Local guidelines on seeking consent for research with Indigenous populations. (XLSX 35 kb)

## Abbreviations

AAND: Aboriginal affairs and northern development
AH&MRC: Aboriginal health and medical research council
AIATSIS: Australian indigenous aboriginal and torres strait islander studies
BC: British Columbia
CBD: Secretariat of the convention of biological diversity
CIHR: Canadian institute of health research
CINAHL: Cumulative index for nursing and allied health literature
CIOMS: Council for international organisations of medical science
CLC: Central land council
CRC: Cooperative research centre, torres strait
CRIAH: Coalition for research to improve aboriginal health
CSIRO: Commonwealth scientific and industrial research organisation
DK&CRC: Desert knowledge and cooperative research centre
EMBASE: Excerpta medical database
ERIC: Educational resources information centre
HRC: Health research council, New Zealand
HIV: Human immunodeficiency virus
IIC: Informit indigenous collection
IHS IRB: Indian health service institutional review board
KLC: Kimberley land council
KLC IP & TKP: Kimberley land council and traditional knowledge policy
Medline: Medical literature analysis and retrieval system online
NEAC: National ethics advisory committee
NHMRC: National health and medical research council
NIH: National institute of health
NSW: New South Wales
NT: Northern Territory
NZ: New Zealand
PRISMA-P: Preferred reporting items for systematic review and meta-analysis protocols
PWG: Pūtaiora writing group
MeSH: Medical subject headings
REAC: Research access committee
SNC: Six Nations council
TEC: Tribal epidemiology centres
TMR: Triple media recording
UN: United Nations
UNDRIP: United Nations declaration on the rights of indigenous peoples
UoVic: University of Victoria
USA: United States of America
Vic: Victoria
WHO: World Health Organisation
WIPO: World intellectual property
WMA: World Medical Association
WMA DOH: World Medical Association Declaration of Helsinki
WIPO: World intellectual property organisation
